# Preventive Activity of Paroxetine Against the Formation of Mixed Biofilms of *Candida albicans* and *Staphylococcus aureus* on Peripheral Venous Catheters

**DOI:** 10.1007/s00284-026-05088-6

**Published:** 2026-10-01

**Authors:** Lívia Gurgel do Amaral Valente Sá, Érica Rayanne Mota da Costa, Maria Janielly Castelo Branco Silveira, Vitória Pessoa de Farias Cabral, Henrique Santos Carneiro, Leilson Carvalho de Oliveira, Thais Lima Ferreira, Daniel Sampaio Rodrigues, Lara Elloyse Almeida Moreira, Vitória Monteiro Matos, Vitória Girão dos Santos, João Batista de Andrade Neto, Hélio Vitoriano Nobre Júnior, Cecília Rocha da Silva

**Affiliations:** 1 School of Pharmacy, Laboratory of Bioprospection in Antimicrobial Molecules (LABIMAN), Federal University of Ceará, Fortaleza, CE Brazil; 2 Drug Research and Development Center (NPDM), Federal University of Ceará, Fortaleza, CE Brazil; 3 Christus University (UNICHRISTUS), Fortaleza, CE Brazil

## Abstract

Catheters are often sources of bloodstream infections since they promote the development of polymicrobial biofilms composed of *Candida albicans* and *Staphylococcus aureus*, which are resistant to conventional treatments. Paroxetine (PRX), a selective serotonin reuptake inhibitor, has demonstrated antibiofilm activity against both *C. albicans* and *S. aureus*. However, studies evaluating PRX’s activity against polymicrobial biofilms remain scarce. Consequently, we investigated the activity of PRX against mixed biofilms of *C. albicans* and *S. aureus*, as well as its potential to prevent the formation of such biofilms on peripheral venous catheters. Our findings revealed that PRX not only prevented biofilm formation but also significantly reduced both the viability and biomass of these structures, possibly by disrupting the polysaccharide matrix. When impregnated in peripheral venous catheters, PRX also inhibited the excessive growth of microbial communities, making it a potentially effective agent to modulate the structure and susceptibility of multispecies biofilms, expanding its spectrum of action to more complex clinical scenarios associated with medical devices.

## Introduction

Bloodstream infections (BSIs) are serious health problems that can lead to host mortality, thereby representing a global public health concern [1]. In developed countries, the rate of nosocomial infections is approximately 15%. This rises to 25% in developing nations, overall resulting in the death of nearly 40,000 patients worldwide per year [2]. Intensive care units (ICUs) are particularly conducive to the spread of infections due to the immunocompromised status of patients, the frequent use of antibiotic therapy, and the performance of invasive medical procedures [3].

Among the invasive interventions routinely required in hospital settings is the use of peripheral venous catheters (PVCs), which are estimated to be applied at a frequency nearly 60 times higher than central venous catheters [4]. BSIs originating from catheter use are among the leading causes of sepsis in ICUs, contributing to increased mortality rates, higher hospital costs, and longer hospital stays. These devices create a microenvironment that favors biofilm formation and facilitates microbial adhesion [5].

Hospital-acquired infections associated with catheter use are frequently caused by resistant microorganisms, primarily *Staphylococcus* spp., *Enterococcus* spp., certain Enterobacteriaceae, and yeasts of the genus *Candida* spp. [6]*.* Among these, *S. aureus* stands out as the most frequently isolated bacterium from peripheral catheters, even though it is part of the normal human microbiota [4]. In ICUs, approximately 20% of patients infected with methicillin-resistant *S. aureus* (MRSA) progress to death [7]. Indeed, *S. aureus* was responsible for over 1 million deaths worldwide in 2019 and was the bacterium most associated with mortality in 135 countries [8].

It is estimated that 626,081 cases of candidemia occur annually (8.05 per 100,000 inhabitants) [9]. Approximately 20% of BSIs caused by *C. albicans* are polymicrobial, with *S. aureus* being the third most commonly isolated co-pathogen. These microbial associations are common in diseases affecting the skin and mucosa, which are sites where these microorganisms are typically commensal. Furthermore, they are also involved in infections related to medical device use, in which they tend to organize into biofilm structures [10].

Biofilm formation is a shared virulence factor among resistant microorganisms, including MRSA and *Candida* spp., complicating treatment due to the persistence of infection. As such, biofilms pose a significant challenge to the management of infections caused by resistant pathogens [11].

Furthermore, biofilms are often composed of a mixed form of bacteria, fungi, ans/or viruses, forming polymicrobial communities. As a result**,** a polymicrobial composition exhibits greater resistance to antimicrobials and disinfectants compared to a monospecies biofilm [12]. Increased resistance was reported by Nabb et al*.*, (2019) [13], who compared the tolerance of sessile cell cultures of *S. aureus* with polymicrobial cultures of *S. aureus* and *C. albicans*, observing that *S. aureus* in the presence of *C. albicans* demonstrated a 10- to 100-fold greater tolerance to treatments with oxacillin, vancomycin, ciprofloxacin, delafloxacin and rifampin.

Therefore**,** catheter-associated BSIs require targeted strategies for managing medical devices, aiming to reduce or prevent microbial adhesion to the surface, thereby preventing biofilm formation [14]. Given this critical issue, it is imperative to invest in the development of new treatments to combat polymicrobial infections caused by *C. albicans* and *S. aureus*.

In this context, drug repositioning is a therapeutic redirection strategy aimed at identifying new applications for medications already approved in clinical trials for other purposes [15]. For instance, there are reports in the literature regarding the antimicrobial activity of selective serotonin reuptake inhibitors (SSRIs) [16], such as fluoxetine [17, 18], sertraline [19, 20] and paroxetine (PRX) [21, 22], against *S. aureus* and *Candida* spp., including their biofilm forms.

PRX, in particular, has already demonstrated activity against biofilms of *C. albicans* and *S. aureus* [16, 22]. However, studies evaluating PRX’s activity against polymicrobial biofilms formed by these microorganisms remain scarce. Thus, we investigated PRX’s activity against mixed biofilms of *C. albicans* and *S. aureus*, as well as assessed its potential to prevent the formation of such structures on PVCs.

## Materials and Methods

### Microorganisms and Drug

Two clinical isolates of methicillin-resistant *S. aureus* (MRSA) and two of *C. albicans* were used, selected based on their high biofilm-forming capacity to ensure robust and reproducible in vitro biofilm models for the evaluation of PRX activity, which were obtained from the collection of the Laboratory for Bioprospecting of Antimicrobial Molecules (LABIMAN), belonging to Federal University of Ceará (UFC). Since these were pre-existing and fully anonymized historical isolates, formal ethical approval was not required. The minimum inhibitory concentration (MIC) was previously determined by Cabral et al*.* (2023) [21] for *S. aureus* and by Costa Silva et al*.* (2017) [16] for *C. albicans*. Accordingly, the MIC of PRX for the combination was determined to be 64 µg/mL (*C. albicans* 1 + MRSA 1) and 80 µg/mL (*C. albicans* 2 + MRSA 2). The susceptibility of the strains was assessed according to the M100-S31 protocol [23] of the Clinical and Laboratory Standards Institute for oxacillin against *S. aureus*, and the M27-S4 protocol [24] for fluconazole against *C. albicans*.

PRX hydrochloride was purchased from Galena Química e Farmacêutica Ltda. (Brazil) and diluted in dimethyl sulfoxide (DMSO; ≤ 2.5%).

### Biofilm Assays

#### Mixed Biofilm Cultivation

The protocol was carried out according to She et al*.*, (2020) [25] and Silva et al*.*, (2024) [26], with modifications. The selected strains were classified as strongly adherent based on biomass assessment using the 0.1% crystal violet assay [27]. Twenty-four-hour cultures of *C. albicans* and MRSA were collected. Subsequently, *C. albicans* was cultured in yeast nitrogen base (YNB) broth (Difco, Becton Dickinson and Sparks, MD, USA) supplemented with glucose (100 mM) (Isofar, Duque de Caxias, RJ, Brazil), and MRSA in tryptic soy broth (TSB) (Kasvi, Pinhais, PR, Brazil) supplemented with 2% glucose. Cultures were centrifuged (2500 rpm for 5 min) and washed three times with saline solution (0.85% NaCl). Inocula were then prepared at 0.5 McFarland standard (1 × 10⁶ CFU/mL) for *C. albicans* and 1.0 McFarland standard (3 × 10⁸ CFU/mL) for MRSA in TSB medium.

#### Biofilms in Microplates

Two combinations of fluconazole-resistant *C. albicans* and MRSA were used in the assays. In the microplate biofilm assays, the PRX concentrations evaluated corresponded to the MIC, 4×MIC and 8×MIC. For the biofilm formation prevention tests, 50 µL of *C. albicans* inoculum and 50 µL of MRSA inoculum were added to 96-well plates, along with 100 µL of the different PRX concentrations diluted in the culture medium. These plates were incubated for 48 h at 35 °C. Wells for growth control (TSB + inoculum) and sterility control (TSB) were also included.

In the mature biofilm assays, *C. albicans* (50 µL) and MRSA (50 µL) inocula were incubated with 100 µL of culture medium (TSB + 2% glucose) for 48 h at 35 °C. After biofilm formation, the wells were carefully washed with saline solution to remove non-adherent cells. Subsequently, 200 µL of PRX solution was added to the respective wells, followed by incubation of the plates for 24 h.

After incubation, the biofilms were washed with saline. Metabolic activity was assessed using the colorimetric assay with 3-(4,5-dimethyl-2-thiazolyl)− 2,5-diphenyl-2H-tetrazolium bromide (MTT) (Sigma-Aldrich). Biomass quantification was performed according to Stepanović et al*.* (2000) [27] using the 0.1% crystal violet assay (Sigma-Aldrich). Absorbance analysis was conducted at 570 nm using a SmartReader™ 96 microplate reader (Accuris Instruments, NJ, USA).

Extracellular matrix (ECM) quantification of the biofilm was performed using the colorimetric safranin assay (Sigma-Aldrich). The protocol was carried out as modified from Costa-Orlandi et al*.* (2014) [28] and Ramos et al*.* (2020) [29]. A volume of 50 µL of 0.1% safranin (diluted in saline) was added to each well for 15 min. Each well was then washed twice with saline solution, followed by the addition of 30% acetic acid to solubilize the dye. Finally, 100 µL of the supernatant was transferred to a new 96-well plate. Absorbance was quantified using the SmartReader™ 96 microplate reader at 570 nm.

##### Data Analysis

Each experiment was performed three times on separate days. Results were compared to determine statistical significance (p<0.05) using analysis of variance (ANOVA), followed by the Tukey test. Data were compiled using GraphPad Prism version 6.0 (GraphPad Software).

#### Catheter Biofilms

The isolates *C. albicans* 1 and MRSA 1 were used as representative strains.

##### PVC Impregnation with PRX

PVC catheters were cut into 0.5 cm fragments (Jelco 18G Descarpack). The method was adapted from Fisher et al*.* (2015) [30] and Nair et al*.* (2016) [31]. PRX was diluted in chloroform (Dinâmica), and the fragments were submerged for 24 h. Control catheter fragments were submerged in chloroform only. The PVC fragments were then placed in sterile Petri dishes and incubated for 24 h at 35 °C to ensure complete drying. Subsequently, growth control fragments were incubated with the inoculum, while sterility control fragments were incubated with culture medium only. For the catheter biofilm prevention assays, the PRX concentration tested was 10 mg/mL (approximately 1% *w/v*), based on a previously reported catheter coating study [32].

##### Data Analysis

All assays were performed in triplicate. Data were analyzed using a one-tailed paired Student *t*-test (p<0.05) with GraphPad Prism version 5.0.

##### Scanning Electron Microscopy (SEM) of Catheter Biofilm

The assay was conducted according to Silva et al*.* (2024) [26], with some modifications. Adherent cells on the catheter fragments were treated with PRX at a concentration of 10 mg/mL. Following this initial step, samples were fixed with 2.5% glutaraldehyde (Sigma-Aldrich) and incubated overnight at 4 °C. The catheter fragments were then washed twice with a 0.1 M cacodylate buffer solution (Electron Microscopy Sciences) containing 0.1% Alcian blue (Sigma-Aldrich), followed by dehydration using increasing concentrations of ethanol. Subsequently, the samples were treated with hexamethyldisilazane (HMDS) until complete evaporation of the reagent. For microscopic analysis, the fragments were coated with a 10 nm layer of gold (Emitech, Uckfield, United Kingdom; Q150T) and examined using an FEI Inspect S50 scanning electron microscope (FEI Company, MA, USA).

## Results

### PRX Demonstrated the Ability to Reduce the Viability of Mixed Biofilms Formed by *C. albicans* and MRSA

#### Mature Biofilms

**Fig. 1:**
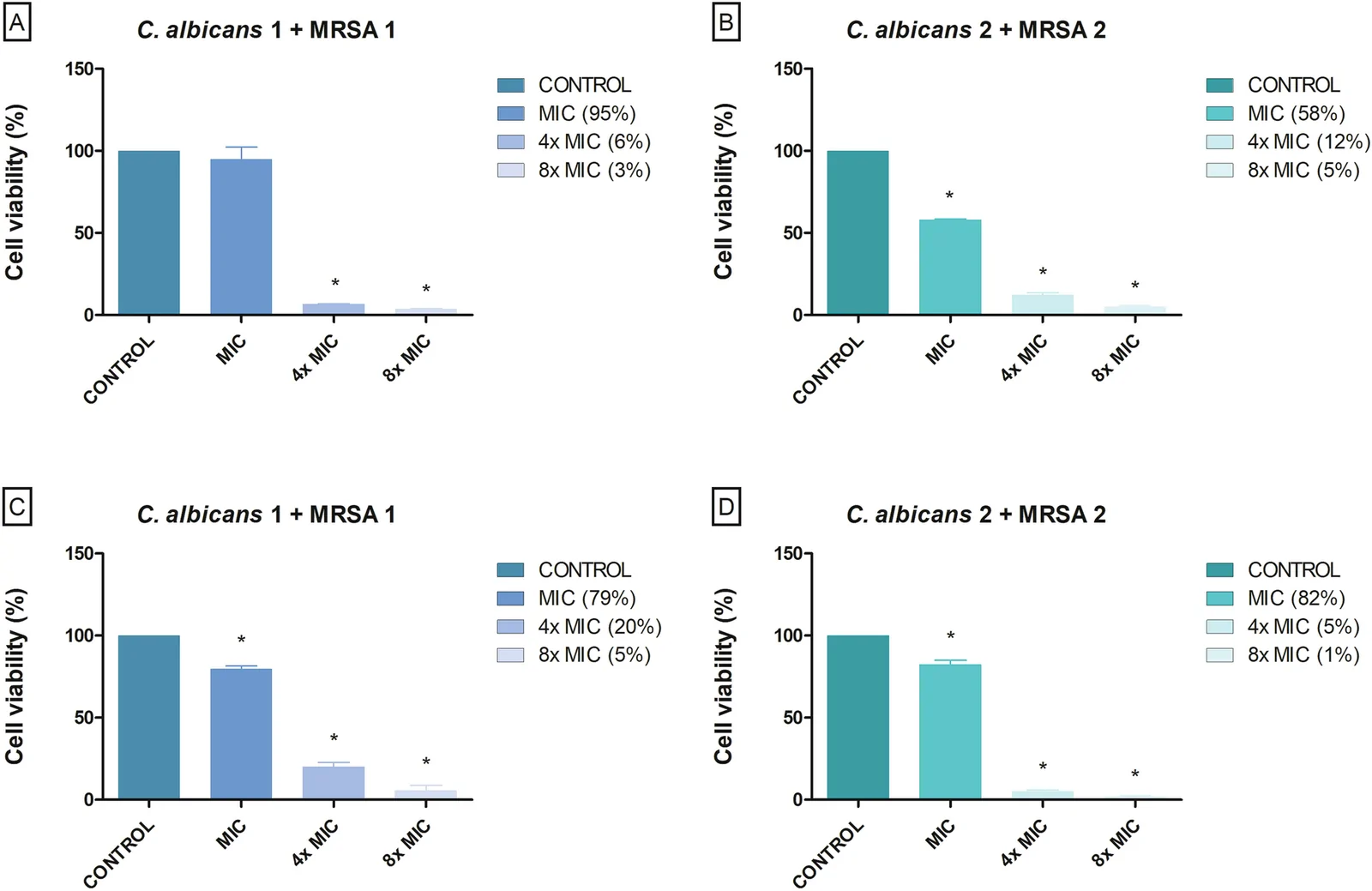
Effect of PRX on the cellular viability of developing (A and B) and mature (C and D) biofilms of *C. albicans* and MRSA, determined by the MTT reduction assay. CONTROL: Biofilm formed without drug addition, corresponding to 100% cell viability. *C. albicans* 1 + MRSA 1 (**A**) – MIC: 64 µg/mL; 4× MIC: 256 µg/mL; 8× MIC: 512 µg/mL. *C. albicans 2* + MRSA 2 (**B**) – MIC: 80 µg/mL; 4× MIC: 320 µg/mL; 8× MIC: 640 µg/mL. *C. albicans* 1 + MRSA 1 (**C**) – MIC: 64 µg/mL; 4× MIC: 256 µg/mL; 8× MIC: 512 µg/mL. *C. albicans* 2 + MRSA 2 (**D**) – MIC: 80 µg/mL; 4× MIC: 320 µg/mL; 8× MIC: 640 µg/mL. PRX: paroxetine. MRSA: methicillin-resistant *S. aureus*. MTT: 3-(4,5-dimethyl-2-thiazolyl)− 2,5-diphenyl-2H-tetrazolium bromide. (*) p < 0.05: Results were compared to the growth control. Statistical significance was determined using ANOVA followed by the Tukey test

In the association between *C. albicans* 1 and MRSA 1, a significant reduction in the viability of the mature biofilms was observed at the highest PRX concentrations tested. The concentration equivalent to the MIC reduced viability by 5%, while the 4× MIC and 8× MIC concentrations promoted more substantial reductions, of approximately 94% and 97%, respectively. In the association between *C. albicans* 2 and MRSA 2, reductions in viability were detected at all concentrations evaluated: the MIC reduced viability by approximately 50%, the 4× MIC by around 88%, and the 8× MIC by 95% (Fig. 1).

#### Biofilm Formation

During the biofilm development phase, PRX was able to reduce microbial viability at all concentrations tested. At the concentration equivalent to the MIC, approximately 20% inhibition of biofilm growth was observed in both associations (*C. albicans* 1 + MRSA 1 and *C. albicans* 2 + MRSA 2). In the *C. albicans* 1 + MRSA 1 association, PRX at the 4× MIC promoted a reduction of about 80% in biofilm growth, while at the 8× MIC concentration, inhibition reached approximately 95%. Conversely, in the *C. albicans* 2 + MRSA 2 association, PRX exhibited greater efficacy: the 4× MIC inhibited approximately 95% of the biofilm, and the 8× MIC achieved up to 99% inhibition (Fig. 1).

### PRX Disrupted the Structural Integrity of Mixed Biofilms Formed by *C. albicans* and MRSA

#### Mature Biofilms

**Fig. 2:**
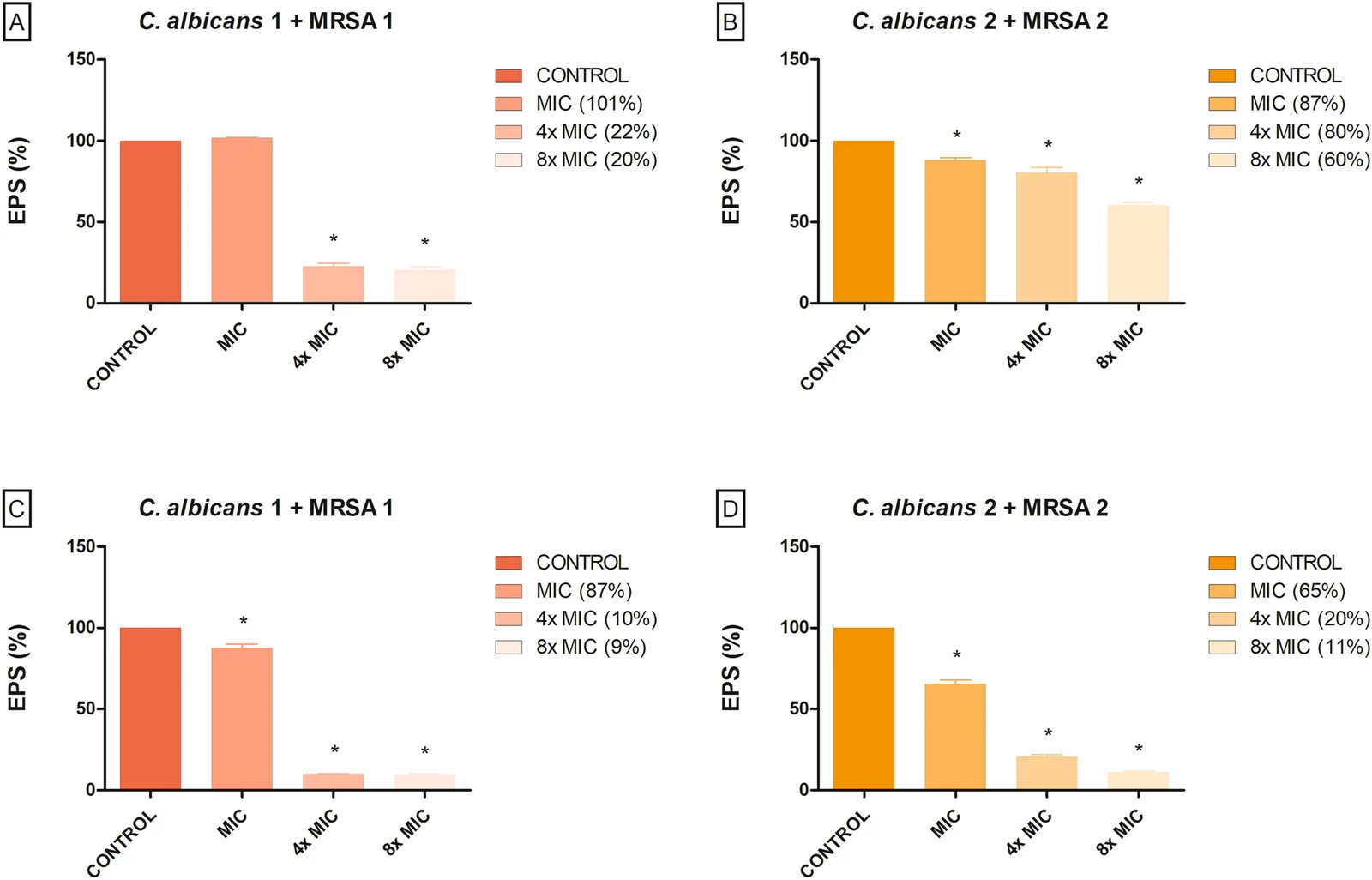
Effect of PRX on the ECM structure of developing (A and B) and mature (C and D) biofilms formed by *C. albicans* and MRSA, as determined by the safranin staining assay. CONTROL: Biofilm formed without drug addition, corresponding to 100% viability. *C. albicans* 1 + MRSA 1 (**A**) – MIC: 64 µg/mL; 4× MIC: 256 µg/mL; 8× MIC: 512 µg/mL. *C. albicans* 2 + MRSA 2 (**B**)—MIC: 80 µg/mL; 4× MIC: 320 µg/mL; 8× MIC: 640 µg/mL. *C. albicans* 1 + MRSA 1 (**C**) – MIC: 64 µg/mL; 4× MIC: 256 µg/mL; 8× MIC: 512 µg/mL. *C. albicans* 2 + MRSA 2 (**D**) – MIC: 80 µg/mL; 4× MIC: 320 µg/mL; 8× MIC: 640 µg/mL. PRX: paroxetine. ECM: extracellular matrix. MRSA: methicillin-resistant *S. aureus*. (*) p < 0.05: Results were compared to the growth control. Statistical significance was determined using ANOVA followed by the Tukey test

In mature biofilms, PRX exhibited significant activity against the cellular matrix of the *C. albicans* 1 + MRSA 1 association at the highest concentrations tested. The concentration equivalent to the MIC did not affect the biofilm structure. However, the 4× MIC was able to disrupt approximately 78% of the matrix, while the 8× MIC resulted in a slightly greater destruction (80%). In the *C. albicans* 2 + MRSA 2 association, PRX demonstrated overall higher efficacy, significantly reducing the biofilm matrix at all evaluated concentrations. Treatment at the MIC caused 13% disorganization of the biofilm matrix, while the 4× MIC led to 20% disruption, and notably the 8× MIC compromised approximately 40% of the microbial structure (Fig. 2).

#### Biofilm Formation

During the initial phase of biofilm development, PRX exhibited effective antimicrobial activity against both associations tested. In the *C. albicans* 1 + MRSA 1 combination, all concentrations showed activity against the cellular matrix, with the MIC reducing the structure by 13%, while 4× MIC and 8× MIC led to matrix disruptions of 90% and 91%, respectively. In the *C. albicans* 2 + MRSA 2 association, activity was also observed at all tested doses, with the MIC reducing the matrix by 35%, and 4× MIC and 8× MIC achieving reductions of 80% and 89%, respectively (Fig. 2).

### PRX Promoted Reduction of Cellular Biomass in Mixed Biofilms Formed by *C. albicans* and MRSA

#### Mature Biofilms

**Fig. 3:**
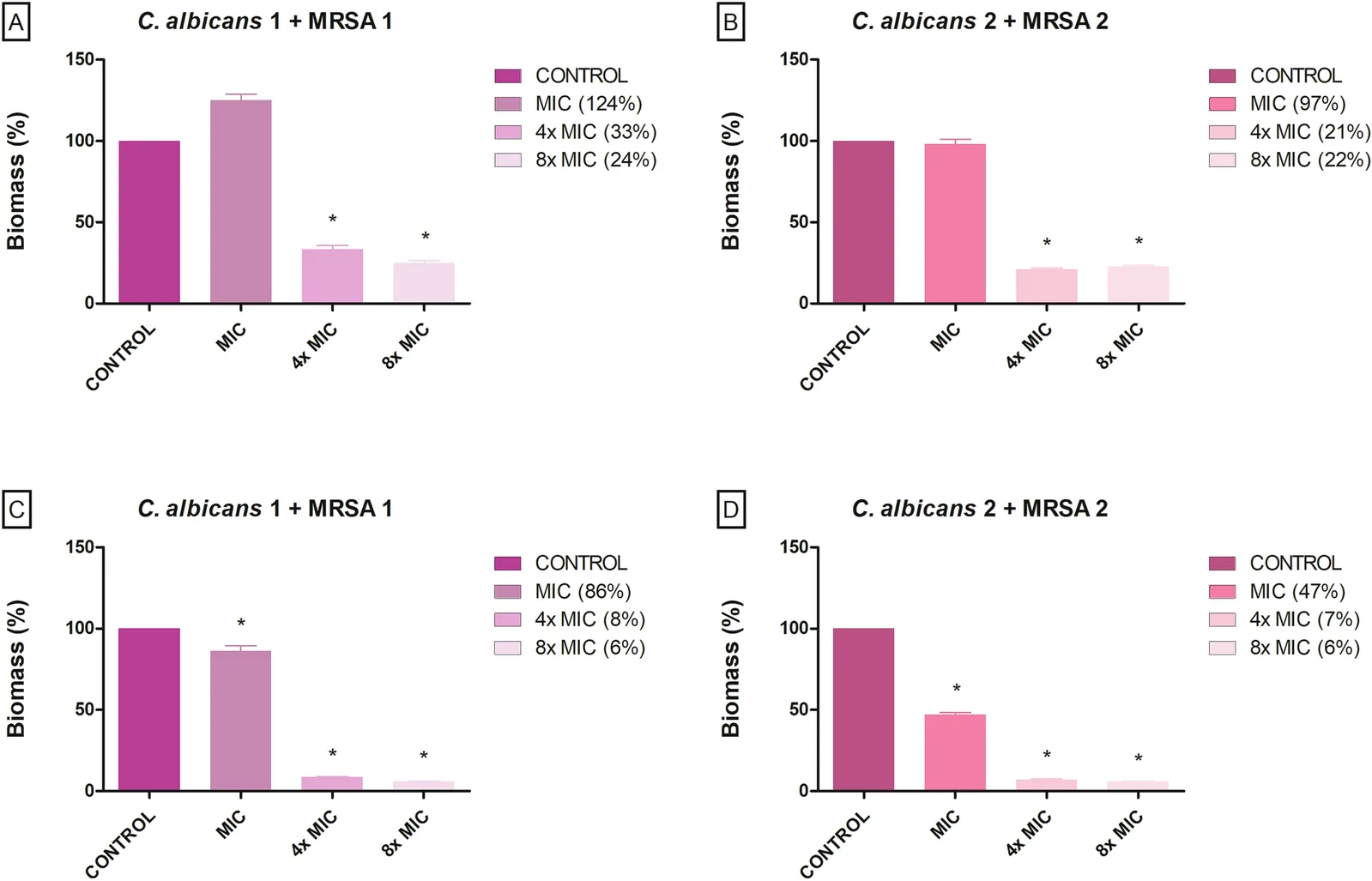
Effect of PRX on the biomass of developing (A and B) and mature (C and D) biofilms formed by *C. albicans* and MRSA, as determined by the crystal violet staining assay. CONTROL: Biofilm formed without drug addition, corresponding to 100% viability. *C. albicans* 1 + MRSA 1 (**A**) – MIC: 64 μg/mL; 4× MIC: 256 µg/mL; 8× MIC: 512 µg/mL. *C. albicans* 2 + MRSA 2 (**B**)—MIC: 80 µg/mL; 4× MIC: 320 µg/mL; 8× MIC: 640 µg/mL. *C. albicans* 1 + MRSA 1 (**C**) – MIC: 64 µg/mL; 4× MIC: 256 µg/mL; 8× MIC: 512 µg/mL. *C. albicans* 2 + MRSA 2 (**D**) – MIC: 80 µg/mL; 4× MIC: 320 µg/mL; 8× MIC: 640 µg/mL. PRX: paroxetine. MRSA: methicillin-resistant *S. aureus*. (*) p < 0.05: Results were compared to the growth control. Statistical significance was determined using ANOVA followed by the Tukey test

In evaluating PRX’s effect on biofilm biomass, the drug demonstrated significant efficacy at higher doses of both associations. In the *C. albicans* 1 + MRSA 1 combination, the MIC did not induce any reduction, whereas the 4× MIC (67%) and 8× MIC (76%) concentrations led to notable biomass decreases. In the *C. albicans* 2 + MRSA 2 association, the MIC caused minimal biomass reduction, while the 4× MIC (79%) and 8× MIC (78%) concentrations resulted in more substantial decreases (Fig. 3).

#### Biofilm Formation

Regarding the biomass of developing biofilms, PRX demonstrated significant efficacy at all tested concentrations. In the *C. albicans* 1 + MRSA 1 association, the MIC promoted a 14% reduction in biomass, while the 4× MIC and 8× MIC concentrations resulted in respective reductions of 92% and 94%. In the second association, *C. albicans* 2 + MRSA 2, the MIC already led to a 53% reduction, and the 4× MIC and 8× MIC concentrations resulted in reductions of 92% and 94%, respectively (Fig. 3).

### PRX Demonstrated Antimicrobial Potential by Reducing Microbial Load on Catheters Colonized by Mixed Biofilms of *C. albicans* and MRSA

**Fig. 4:**
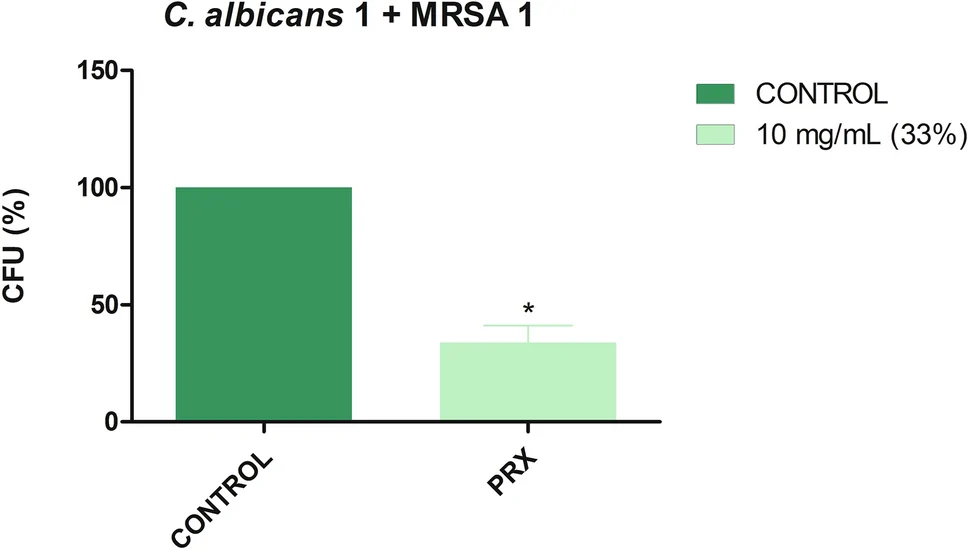
Effect of PRX on the reduction of microbial load (CFU/mL) in developing biofilms of *C. albicans* and MRSA, as determined by colony counting. CONTROL: Biofilm formed without drug addition, corresponding to 100% cell viability. PRX: 10 mg/mL. PRX: paroxetine. MRSA: methicillin-resistant *S. aureus*. (*) p < 0.05: Results were compared to the growth control. Statistical significance was determined using Student’s t-test

Figure 4 illustrates the preventive activity of PRX impregnated in catheters against mixed biofilms of *C. albicans* and MRSA. At a concentration of 10 mg/mL, the compound was able to reduce the microbial load adhered to the device by 67% compared to the growth control, thereby highlighting its antimicrobial potential to prevent colonization by these pathogens.

### PRX Induced Morphological Alterations in Mixed Biofilm Cells of *C. albicans *and MRSA Adhered to Catheter Fragments

**Fig. 5:**
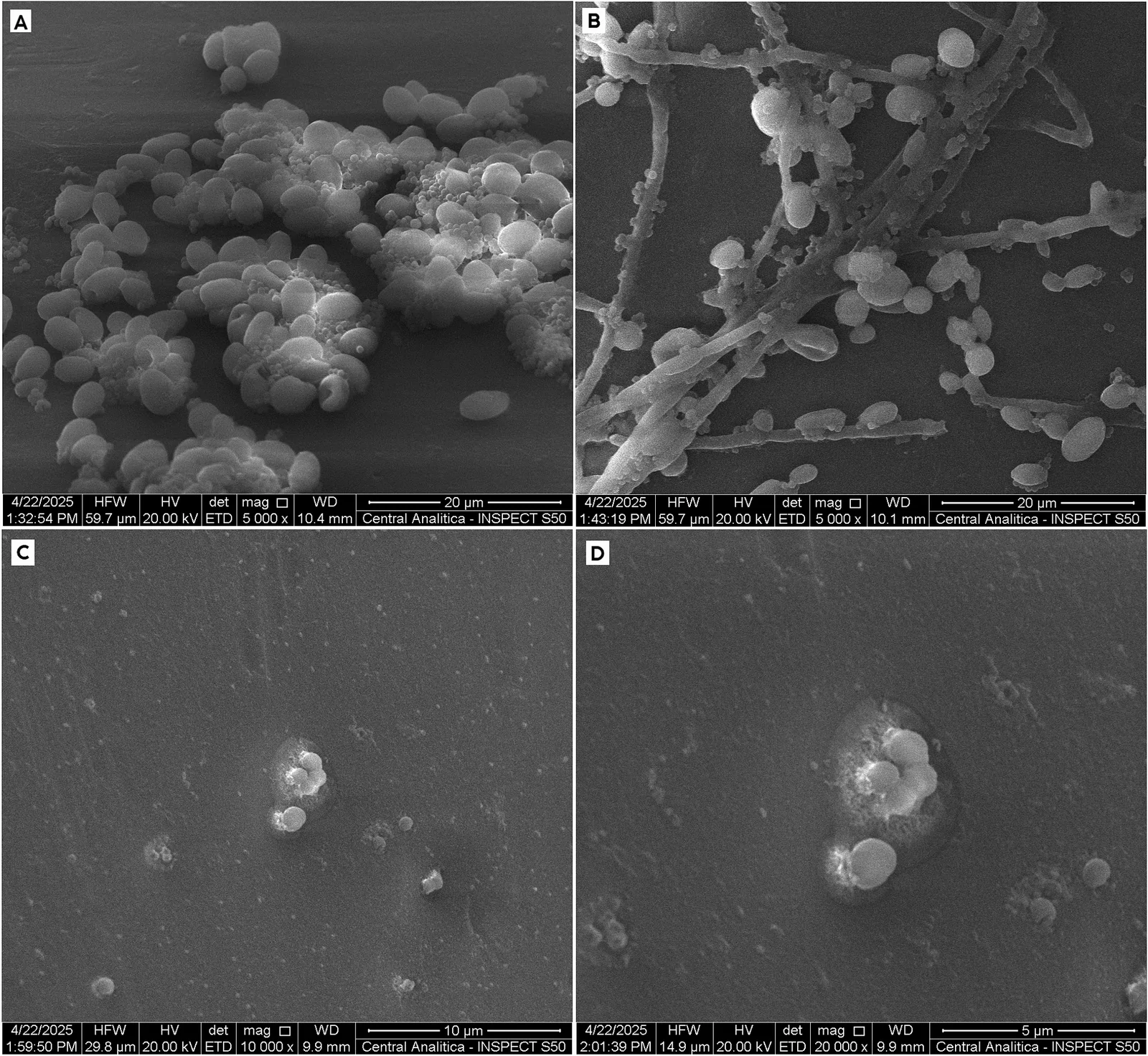
Effect of PRX on the morphology of developing biofilm cells of *C. albicans* and MRSA, observed by SEM. (A and B): Positive control—morphologically intact cocci, yeast cells, and budding hyphae. (C and D): Reduction in cell density and morphological alterations suggestive of membrane damage caused by PRX at a concentration of 10 mg/mL. Images **A** and **B** were obtained at 5,000× magnification (Scale bar: 20 µm); image **C** at 10,000× magnification (Scale bar: 10 µm); and image **D** at 20,000× magnification (Scale bar: 5 µm). PRX: paroxetine. MRSA: methicillin-resistant *S. aureus*. SEM: scanning electron microscopy

To investigate morphological changes induced by PRX treatment, we used SEM of the biofilms formed by *C. albicans* and MRSA adhered to peripheral catheter fragments, as illustrated in Fig. 5. Images A and B correspond to the positive control, showing cocci clustered in grape-like formations, yeasts and hyphae with preserved morphology, and intact cell surfaces. Images C and D, however, illustrate the action of PRX on the biofilm adhered to the catheter fragments, revealing a marked reduction in cell density compared to the control, in addition to the presence of cells with evident morphological damage.

## Discussion

Species of the genera *Staphylococcus* and *Candida* rank among the primary etiologic agents isolated in catheter-related BSIs. In this context, coinfection involving these species establishes a synergistic relationship that enhances their virulence, contributing to poorer clinical outcomes and increased resistance to conventional treatment [12, 33]. These findings underscore the importance of exploring novel therapeutic approaches and more effective control strategies.

In this study, we evaluated the activity of PRX against mixed biofilms formed by MRSA and FLC-resistant *C. albicans*. In the MTT cell viability assay, the antidepressant significantly reduced (P < 0.05) the viability of mature biofilms in both associations, with more pronounced activity at concentrations equivalent to 4× and 8× MIC, where reductions reached approximately 90%. Additionally, PRX demonstrated potent preventive action against biofilm formation, with inhibition rates exceeding 80% at the highest concentrations tested, indicating dose-dependent behavior.

Although to date, no published studies have investigated the effect of PRX on mixed biofilms of *Candida* and *S. aureus*, these findings are consistent with literature reports describing PRX’s ability to inhibit the growth of planktonic cells of various *Candida* species [16, 34], as well as significantly reduce the viability of MRSA biofilms, with over 90% reduction in mature biofilms and more than 90% prevention of early biofilm development [22]. Furthermore, fluoxetine, a functional analog of PRX, is also described in the literature as an antimicrobial agent, exhibiting significant activity against mature biofilms of *Candida* species [16].

The drug’s effect on biomass reduction was also assessed in this study. In mature polymicrobial biofilms, a decrease of approximately 70% in biomass was observed following PRX treatment. In developing biofilms, the reduction reached around 90% in both tested associations. In line with these findings, Viveiro et al*.* (2024) [35] demonstrated that PRX is also capable of reducing biomass by approximately 60% in mature *Cryptococcus gattii* biofilms, reinforcing its potential as an antibiofilm agent against diverse microorganisms.

The ECM is a critical component of biofilms, functioning as a protective structure that surrounds microbial cells and contributes to surface adhesion, nutrient retention and tolerance to antimicrobial agents [12, 36]. In polymicrobial biofilms, the synergistic interaction between different species promotes increased production of exopolysaccharides, proteins, and extracellular DNA, resulting in a more robust and complex matrix [37, 38].

In communities formed by *S. aureus* and *C. albicans*, for instance, the presence of the yeast stimulates matrix production by *S. aureus*, thereby enhancing the structural stability and tolerance of the biofilm under adverse conditions [39, 40]. In light of this, we investigated the potential of PRX against the ECM using the safranin staining assay. The results revealed that the antidepressant was capable of reducing the cellular structure of the *C. albicans* 1 + MRSA 1 combination by up to 80%, in addition to inhibiting approximately 90% of biofilm formation in both associations.

Previously, Baraka et al*.* (2025) [41] reported the activity of PRX against *Pseudomonas aeruginosa* biofilms using the quantitative Congo red assay (CRA). In that study, a 68.18% reduction in biofilm formation was observed. CRA, originally a textile dye, is widely used in microbiology to detect ECM overproduction by the analyzed strains, since it binds to polysaccharides present in the matrix, allowing inference of its abundance [42]. In this context, the findings described by Baraka et al*.* (2025) [41] support our results by demonstrating PRX-induced reduction of the ECM, thereby reinforcing its role in biofilm disruption.

Biofilm formation on/in medical devices is extensively reported in the literature and represents a significant clinical concern [43]. In this scenario, the presence of polymicrobial biofilms involving *C. albicans* and *S. aureus* poses an even greater challenge. The ability of *C. albicans* to form hyphae creates a filamentous structure that serves as an anchoring point for *S. aureus* cells (Fig. 5B), promoting synergistic interaction between the species. This cooperation results in a denser, more organized, and resistant biofilm, hindering the action of antimicrobial agents and contributing to persistent infections associated with invasive devices [40, 44].

The efficacy of PRX against monomicrobial biofilms fixed on catheters has already been demonstrated in the literature. Farias Cabral et al*.* (2025) [22] observed a 44.81% reduction in *S. aureus* biofilm formation on catheters treated with the drug, along with morphological alterations revealed by SEM. Building on this approach, we evaluated the action of PRX on polymicrobial biofilms adhered to peripheral venous catheters (PVCs). The data obtained revealed a 67% reduction in microbial structure compared to the control group, indicating significant PRX activity in disrupting the cooperation between the constituent organisms, thus compromising the functional integrity of the biofilm and expanding the drug’s applicability against more complex microbial communities.

It is noteworthy that a concentration of 10 mg/mL was used in a system in which PRX was applied as a coating. In this context, the system performance is associated not only with the amount of compound, but also with the characteristics of the material and the drug–surface interaction, as previously suggested in the literature [45]. These findings therefore indicate the possible existence of an optimal concentration range dependent on the biomaterial–drug system, reinforcing the need for specific optimization for each application. Additionally, in this type of local delivery approach on biomaterial surfaces, studies with catheters coated with antimicrobial agents have demonstrated the absence of significant cytotoxicity in cellular models, further supporting the feasibility and potential safety of this strategy [46, 47].

Observations obtained through SEM visually demonstrated PRX’s ability to reduce the dense structure formed by microbial interactions, particularly highlighting the decrease in hyphal projections that serve as anchoring points for the bacteria. These findings align with the report by Lass-Flörl et al*.* (2003) [48], in which sertraline was shown to inhibit hyphal formation in *Candida* species. Given that both compounds belong to the same pharmacological class, it is plausible that PRX shares a similar mechanism of action, interfering with organism adhesion to the device and directly impacting biofilm architecture.

Moreover, the structural conformation of the biofilm, where *S. aureus* is embedded within a dense fungal ECM, directly contributes to its extreme tolerance to conventional antimicrobials, particularly vancomycin [40]. In this context, our findings are significant: PRX induced a substantial reduction in this matrix, suggesting potential weakening of the protective barrier that facilitates antimicrobial resistance. This reinforces its potential as a modulator of biofilm structure and susceptibility, including as an adjuvant in combination therapies with other antimicrobial agents.

The activity demonstrated by PRX in this scenario suggests that the drug may act on common mechanisms involved in biofilm formation and stability, such as extracellular matrix (ECM) synthesis, which can be attributed to the downregulation of genes associated with initial adhesion and cell–cell anchoring, both essential for the establishment and structural organization of polymicrobial biofilms [49]. In addition, the ability of SSRIs to partition into the lipid bilayer interface promotes changes in membrane physical properties, such as elasticity and curvature, thereby affecting the conformation and functionality of membrane-anchored proteins [50]. Considering that these proteins are directly involved in cell adhesion and in the secretion of ECM components, this effect reinforces the structural impairment of the matrix, since lipid dynamics and microdomain organization are key determinants of polymicrobial biofilm integrity [51].

Additionally, the destabilization of the lipid bilayer may contribute to a potential synergistic interaction with antibiotics, as alterations in membrane properties tend to increase its permeability. In this context, PRX may facilitate the penetration of antimicrobial agents across the cell membrane, enhancing their access to intracellular targets and potentiating their antibiofilm activity [50, 52]. Another mechanism that supports its potential as an adjuvant therapy is the inhibition of efflux pumps, reducing antimicrobial extrusion and promoting their intracellular accumulation. Considering the interplay between efflux pumps and quorum sensing systems, this modulation may also impact intercellular communication, interfering with the coordination and maintenance of the biofilm [52, 53].

It is important to consider some limitations of the present study. The in vitro nature of the experimental model does not fully reproduce the complexity of in vivo biological systems. In addition, the use of a limited number of clinical isolates of *S. aureus* (MRSA) and *C. albicans* may limit the generalizability of the results due to inter-isolate variability. Previous studies have demonstrated that different clinical isolates of both species may exhibit distinct biofilm-forming capacities, virulence traits, antimicrobial susceptibility profiles, and interspecies interaction patterns, which may influence the development and behavior of dual-species biofilms. Therefore, the findings reported here should be interpreted within the context of the isolates evaluated in this study [54, 55]. The study also did not include detailed mechanistic analyses, so the interpretation of the findings is based on observed effects.

Despite this, the obtained results provide relevant evidence of the antibiofilm potential of PRX and reinforce its applicability on biomaterial surfaces. Future studies should include a greater diversity of isolates, as well as mechanistic approaches and in vivo models. In addition, the effects of PRX on dormant microbial subpopulations, such as persister cells, viable but nonculturable (VBNC) cells, and small colony variants (SCVs), remain unknown and warrant further investigation. Likewise, although PRX demonstrated antibiofilm activity at the concentrations evaluated in the present study, its effects at subinhibitory concentrations were not investigated. Considering that sub-MIC levels of certain antimicrobial agents have been reported to enhance biofilm formation in *S. aureus* [56, 57], future studies should assess whether similar responses may occur following PRX exposure in mono- or polymicrobial biofilms. To date, no study has explored PRX as a therapeutic alternative for the treatment of polymicrobial biofilm infections involving *C. albicans* and *S. aureus*. These findings suggest that the activity of PRX may extend beyond planktonic cells and monospecies biofilms, supporting its potential for further investigation in more complex polymicrobial infection models.

## Conclusion

This study demonstrates that PRX exhibits significant activity against polymicrobial biofilms formed by *C. albicans* and *S. aureus*, acting on key mechanisms involved in biofilm formation and stability, such as extracellular matrix production and interspecies interactions. Notably, PRX was able to disrupt the dense biofilm architecture typically associated with increased antimicrobial tolerance in device-related infections. These findings expand the current understanding of PRX’s antimicrobial spectrum, indicating that its activity is not limited to planktonic cells or monospecies biofilms, but may also extend to more complex polymicrobial communities. Overall, PRX emerges as a promising *Candida*te for further investigation as an adjuvant or alternative strategy for managing biofilm-associated infections involving medical devices.

## Data Availability

No datasets were generated or analysed during the current study.
